# Comprehensive Identification and Expression Analysis of the Multidrug and Toxic Compound Extrusion (MATE) Gene Family in *Brachypodium distachyon*

**DOI:** 10.3390/plants13182586

**Published:** 2024-09-15

**Authors:** Sirui Ma, Yixian Guo, Tianyi Zhang, Di Liu, Linna Wang, Ruiwen Hu, Demian Zhou, Ying Zhou, Qinfang Chen, Lujun Yu

**Affiliations:** State Key Laboratory of Biocontrol, Guangdong Provincial Key Laboratory of Plant Stress Biology, School of Life Sciences, Sun Yat-sen University, Guangzhou 510275, China; masr469@163.com (S.M.); guoyx67@mail2.sysu.edu.cn (Y.G.); skybruce091@sina.com (T.Z.); liud47@mail2.sysu.edu.cn (D.L.); wangln45@mail2.sysu.edu.cn (L.W.); hrw807@163.com (R.H.); demychow@163.com (D.Z.); zhouying25@mail.sysu.edu.cn (Y.Z.); chenqf3@mail.sysu.edu.cn (Q.C.)

**Keywords:** MATE, expression profile, phytohormone, *Brachypodium distachyon*

## Abstract

The Multidrug and Toxic Compound Extrusion (MATE) proteins serve as pivotal transporters responsible for the extrusion of metabolites, thereby playing a significant role in both plant development and the detoxification of toxins. The *MATE* gene family within the *Brachypodium distachyon*, which is an important model organism of the Poaceae family, remains largely unexplored. Here, a comprehensive identification and analysis of *MATE* genes that complement *B. distachyon* were conducted. The *BdMATE* genes were systematically categorized into five distinct groups, predicated on an assessment of their phylogenetic affinities and protein structure. Furthermore, our investigation revealed that dispersed duplication has significantly contributed to the expansion of the *BdMATE* genes, with tandem and segmental duplications showing important roles, suggesting that the *MATE* genes in Poaceae species have embarked on divergent evolutionary trajectories. Examination of ω values demonstrated that *BdMATE* genes underwent purifying selection throughout the evolutionary process. Furthermore, collinearity analysis has confirmed a high conservation of *MATE* genes between *B. distachyon* and rice. The cis-regulatory elements analysis within *BdMATEs* promoters, coupled with expression patterns, suggests that *BdMATEs* play important roles during plant development and in response to phytohormones. Collectively, the findings presented establish a foundational basis for the subsequent detailed characterization of the *MATE* gene family members in *B. distachyon*.

## 1. Introduction

Toxic substances in the environment not only affect the growth and development of plants, but also affect the yield of crops. Transporters play an important role in the transport of substances, and toxic substances enter the plant body through transporters [1]. The multidrug and toxic compound efflux (MATE) protein family (also known as detoxifying efflux carrier, DTX) belongs to the multidrug efflux transporter family, which is present in almost all living organisms and has been significantly expanded in plants [2]. The MATE protein family plays a pivotal role during development and in response to various stressors by efficiently managing the processing and detoxification of both exogenous and endogenous toxins [3]. Other well-studied transporter families in plants include the ATP-binding cassette (ABC) family, the major facilitator superfamily (MFS), the resistance–nodulation–division (RND) family, and the small multidrug resistance (SMR) transporters [4]. MATE transporters mediate secondary transport, using Na^+^ or H^+^ electrochemical gradients as the driving force to export toxic substances [5,6].

According to the similarity of MATE amino acids, MATE is divided into NorM, DinF, and eukaryotic MATE (eMATE) subfamilies [5,7]. Both prokaryotic and eukaryotic MATE generally consist of two symmetrical six transmembrane (TM) helixes, and the C-terminal of eMATE and NorM contain conserved acidic residues [6,8]. *MATE* has been identified in prokaryotes and eukaryotes. The first crystal structure (NorM-VC) has been identified in NorM (Vibrio cholerae) and the X-ray crystal structure of *AtDTX14* in Arabidopsis [7,8]. 

There were some studies demonstrated that various MATE transporters were capable of transporting a range of harmful substances or secondary metabolites, which in turn were crucial for managing plant growth and stress responses [9]. Recently, wheat (*Triticum aestivum*) *TaPIMA1* was involved in transporting the anthocyanins and precursors [10]; the mung bean *MIB1* gene could transport tetrabutyl ammonium (TBA) [11]; cucumber *CsMATE1* could transport cucurbitacin C [12]; *NtMATE21* and *NtMATE22* were capable of nicotine or flavonoid [13]; and rice (*Oryza sativa*) *GFD1* was charged for sucrose transporting [14]. Furthermore, a variety of MATE proteins have been implicated in the detoxification of aluminum or the translocation of iron across different plants, including CcMATE35 in pigeon peas [15].

In addition, there were numerous plant organism genomes that were well sequenced, facilitating the discovery of *MATE* genes at the genome-wide levels [9]. In the monocots, there were 211 *MATE* genes in wheat (*Triticum aestivum*) [16]. Furthermore, in the eudicots, 48 *MATE* genes were identified in mung bean (*Vigna radiata* L.) [17]; 85 and 66 *MATE* genes were identified in Rosaceae ‘Dangshansuli’ (*Pyrus bretschneideri Rehd.*) and apple (Malus × domestica Borkh) [18,19], respectively. There were 40–51 *MATE* genes in Cucurbitaceae [20], 35 *MATE* genes in dragon fruit (*Selenicereus undatus*) [21], 63–74 *MATE* genes in mangrove plants [22], and 42–67 *MATE* genes in Solanaceae species [9,23]. Recently, 90 *MATE* genes were identified in the gymnosperm *Torreya grandis* [24].

*Brachypodium distachyon* (L.) *P. Beauv.* (line Bd21) is a widely grown herbaceous plant in the Poaceae family, which contains a small genome and is easy to grow under simple conditions [25,26]. Based on these characteristics of *Brachypodium distachyon*, it is often used as a model plant for functional genomics research of grass crops [27], which belongs to the Pooideae, along with *Triticum aestivum*, *Hordeum vulgare*, and *Avena sativa*.

Here, we made a comprehensive analysis of the *MATE* gene family. In total, 49 *MATE* genes were identified in *Brachypodium distachyon*, 53 genes were identified in rice and 56 genes were identified in *Arabidopsis thaliana*. We divided these *MATE* genes into five groups according to the gene structure of a phylogenetic tree and *MATE*. We found that both tandem repeats and fragment repeats can drive the expansion of *BdMATE* and *OsMATE* gene families, and fragment repetition is the main driving force. In addition, the expression profile data showed that the expression of *BdMATE* was diverse in plant growth and development. The results of subcellular localization showed that there were two different localization of *BdMATE*. Our results provide a basis for further verification of the Poaceae *MATE* gene.

## 2. Results

### 2.1. Identification of MATE Genes in the Brachypodium distachyono Genomes

To achieve a thorough understanding of the *MATE* gene families within *Brachypodium distachyon*, a genome-wide investigation was conducted employing BLASTP, utilizing a set of 56 Arabidopsis AtMATE proteins as the reference queries [28]. Additionally, an HMMER search was implemented to identify the Pfam MATE domain (PF01554). The choice of *Brachypodium distachyon* as the subject of study was motivated by its status as the organism with the most compact genome within the Poaceae family, coupled with its simple living conditions and the availability of well-sequenced and annotated genomes. Subsequent to the initial search, candidate genes were refined based on the localization of the MATE domain and the count of transmembrane domains (TMs), identified through the SMART, InterProscan, and CDD databases. This rigorous approach culminated in the identification of 49 putative *MATE* genes within *Brachypodium distachyon*. In alignment with their chromosomal positions, these genes were sequentially designated as *BdMATE1* through *BdMATE49* (Table S1).

There was a comprehensive description of the 49 *BdMATE* genes, delineating attributes of their encoded proteins such as gene nomenclature, protein length, isoelectric point (pI), computed molecular weight (MW), transmembrane domain (TM) count, and predicted subcellular distribution (Table S1). An exception is noted for the BdMATE37 protein, which exhibits an extended length of 1403 amino acids, contrasting with the 332 to 619 amino acid range observed for the remaining BdMATE proteins. The estimated MW for these proteins varies, with BdMATE37 significantly exceeding the norm at 150.32 kDa, while others fall within the 36.01 to 64.39 kDa. The pI values are predicted to span a spectrum from 5.14 to 10.04. By predicting the transmembrane helices of BdMATE using TMHMM2.0, we found that the number of transmembrane helices of most BdMATEs was predicted to be around 10. Utilizing the WoLFPSORT database, subcellular localization predictions were made for the BdMATE proteins, revealing that the majority, 38 out of 49, are situated in the plasma membrane. Additionally, two BdMATE are predicted to reside in the vacuole, with single representatives in the endoplasmic reticulum, chloroplast, and seven in the nucleus (Table S2).

### 2.2. Phylogenetic Analysis and Structural Characterization of BdMATE Genes

**Figure 1:**
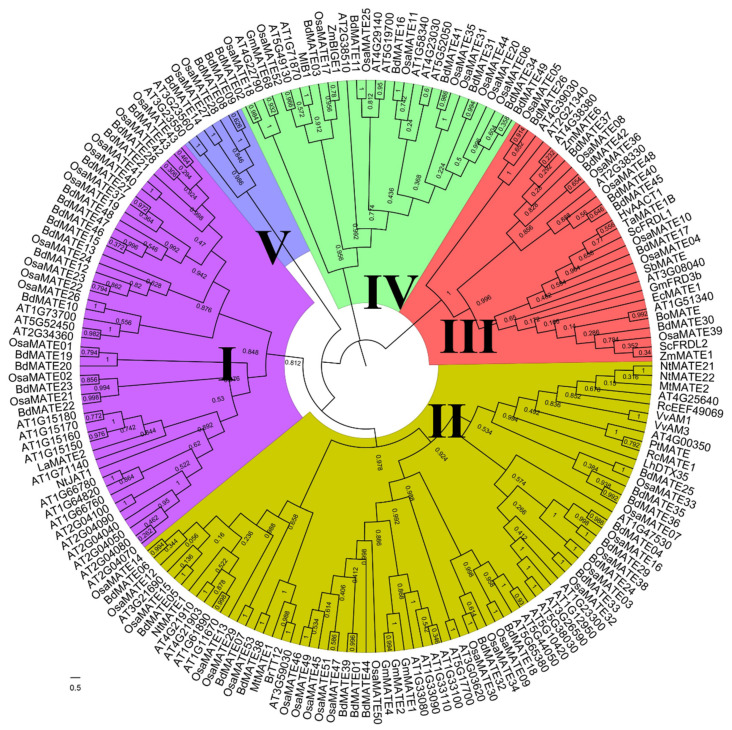
Phylogenetic relationship of 49 *BdMATEs*, 53 *OsMATEs*, 56 *AtMATEs*, and other 30 previously experimental functional identified *MATE* genes. The phylogenetic tree was constructed using MEGA X with the maximum likelihood (ML) method and JTT matrix-based model, which was visualized using the FigTree software (version 1.4.4). *MATE* genes were classified into five distinct groups, as indicated by the different colors.

To explore the phylogeny and evolution of the *MATE* gene family in plants, we constructed a phylogenetic tree using the maximum likelihood method with MegaX software (version X) for 49 *Brachypodium distachyon*, 53 *Oryza sativa*, 56 *Arabidopsis thaliana*, and 30 MATE protein sequences previously experimentally identified [9] (Figure 1). We classified the 188 MATE proteins into 5 groups, Groups I–V, according to the topology of the phylogenetic tree, with high bootstrap values of 84.8, 97.8, 100, 95.6, and 98.6 (Figure 1), respectively, above the significance cutoff of 50. Intra-group bootstrap values were higher than between-group values (Figure 1). The classification was also consistent with the previous study in the *Capsicum annuum*, *Solanum tuberosum*, Rosaceae ‘Dangshansuli’ (*Pyrus bretschneideri Rehd.*), mangrove plants, *Torreya grandis*, and *Citrus sinensis* [9,18,22,24,29]. All five groups contained *Brachypodium distachyon*, *Oryza sativa*, and *Arabidopsis thaliana* MATE proteins, suggesting that the five groups formed before the divergence of the Brassicaceae and Poaceae. The number of MATE proteins associated with each group was uneven in *Brachypodium distachyon*, *Oryza sativa*, and *Arabidopsis thaliana*. Group I and Group II contained the largest number of MATE proteins, with 31 in *Brachypodium distachyon*, 34 in *Oryza sativa*, and 39 in *Arabidopsis thaliana*. *Brachypodium distachyon*, *Oryza sativa*, and *Arabidopsis thaliana* all had the least *MATE* genes in Group V, with three, two, and two genes, respectively.

**Figure 2:**
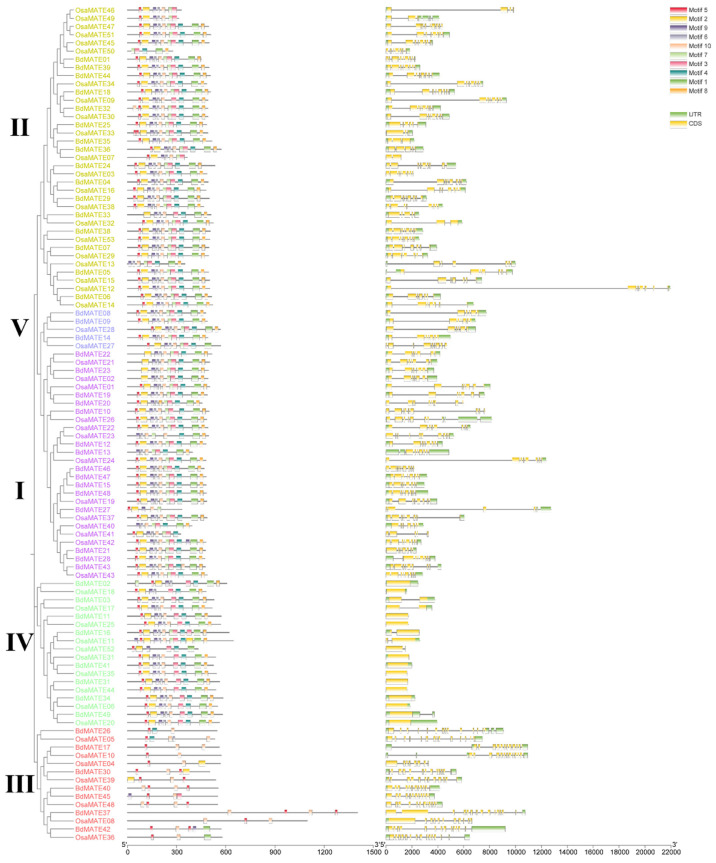
Schematic representation of conserved motifs and gene structure of *Brachypodium distachyon* and *Oryza sativa MATE* family members. Left: phylogenetic tree of 49 *BdMATEs* and 53 *OsMATEs* replotted from Figure 1. Conserved motifs of *B. distachyon* and *O. sativa* MATE proteins. Each color box represents a MATE protein motif identified by MEME motif search tool, and the distribution of the motifs corresponds to their positions. The order of MATE proteins is in accordance with their phylogenetic tree.

To further analyze the structural composition of the *MATE* gene family, we studied the MATE proteins of two species of Poaceae, *Brachypodium distachyon* and *Oryza sativa*. Through MEME, we identified 10 conserved protein motifs (motifs 1–10). At the same time, it was found that the protein arrangement of MATE was roughly consistent with the constructed phylogenetic tree (Figure 1), and all *MATE* genes contained at least one conserved motif (Figure 2 and Figure S1). Furthermore, significant conservation among the motifs was observed for proteins from both species that are classified within the same group, underscored by their diminished E-values and the uniformity in the number and arrangement of motifs. Notably, Groups I, II, IV, and V exhibited analogous protein domain compositions and organizational patterns, which were markedly divergent from those observed in Group III. For instance, while Group III displayed only 2–4 motifs in common, Groups I, II, IV, and V demonstrated a broader range of shared motifs, numbering between 7 and 10 (Figure 2). These findings indicated that Group III MATE proteins may have embarked on a divergent evolutionary path relative to the other groups.

To determine the extent of genomic structural diversity of *MATE* genes, we analyzed the exon–intron organization of the *Brachypodium distachyon* and *Oryza sativa MATE* genes, with the help of the GSDS website. The *MATE* gene family of two species had similar exon–intron structures in the same groups (Figure 2), further validating the classification of *MATE* genes. Group I contained 28 *MATE* genes, of which 25 (89.3%) had 7–9 exons and 26 (92.9%) 0–2 introns; Group II contained 37 *MATE* genes, of which 27 (73.0%) had 7–9 exons and 35 (94.6%) introns; Group III contained 14 *MATE* genes, of which 13 (92.9%) had 11–14 exons and 11 (78.6%) had 1–3 introns; and Group V contained 5 *MATE* genes, with 6–9 exons and 0 or 2 introns (Figure 2). Notably, the 18 *MATE* genes belonging to Group IV had 1–2 exons, suggesting a very different genomic structure for these genes (Figure 2).

Our analysis demonstrated that the functional motifs, intron patterns, and exon–intron structures are very similar or the same among the same group of *Brachypodium distachyon* and *Oryza sativa MATE* genes, which are consistent with the phylogeny. Gene structures among different groups varied greatly, which supported the classification of the *MATE* family members.

### 2.3. Chromosomal Distribution and Duplication of Brachypodium distachyon MATE Genes

**Figure 3:**
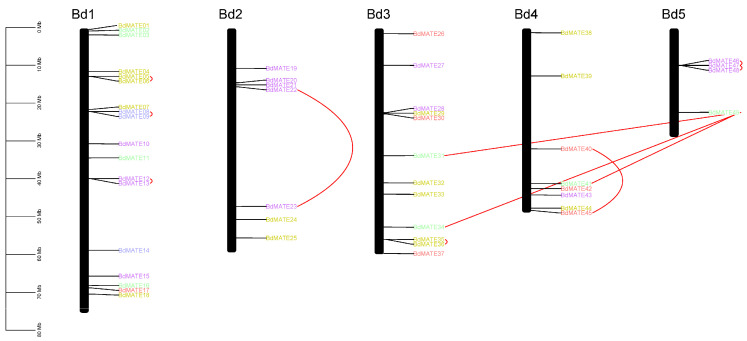
Chromosomal distribution of *Brachypodium distachyon MATE* genes. The number of chromosomes is indicated on the left of each chromosome (vertical bar). The size of chromosomes is indicated by their relative length by using the information from Ensembl database. Tandem-duplicated genes are connected in red arc lines.

To explore the relationship between *Brachypodium distachyon* and *Oryza sativa MATE* genes, we determined their chromosomal locations and whether they originated from gene duplication events. We identified the *BdMATE* gene on all 5 chromosomes, among which there were 18 *BdMATE* genes on chromosome 01, 7 *BdMATE* genes on chromosome 02, 12 *BdMATE* genes on chromosome 03, 8 *BdMATE* genes on chromosome 04, and 4 *BdMATE* genes on chromosome 05 (Figure 3), demonstrating the uneven distribution of genes on the chromosome. In addition, we observed clusters of *BdMATE* genes on chromosome 05 (Figure 3). Similarly, *OsaMATE* genes mapped to all 12 *Oryza sativa* chromosomes, with 2–4 *OsaMATE* genes on chromosomes 01, 02, 04, 07, 08, 09, and 11 (Figure S1).

To further explore the role of *BdMATE* genes duplicate classification during evolution, we used DupGen_Finder to classify its duplicate genes [30], with *Spirodela polyrhiza* as an outgroup. The result showed that there were five pairs of whole-genome duplication genes, three pairs of tandem duplication genes, three pairs of proximal duplication genes, twelve pairs of transposed duplication genes, and forty-one pairs of dispersed duplication genes identified (Table S3), which suggested that dispersed duplication genes accounted for the largest proportion for *BdMATE* genes expansion. In addition, using the tandem-duplicated genes identification criterion [28], along with the MCScanX, we identified 11 *BdMATE* genes in five clusters that correspond to tandem duplication events (Figure 3) and may have contributed to the expansion of the gene family. In total, the union of genes identified by the above two methods was considered as tandem duplication pairs, resulting in six gene pairs (Figure 3). Among these tandem-duplicated genes, three pairs belonged to Group I, two pairs belonged to Group II, and one pair belonged to each of Groups III, V, and IV (Figure 3). In *OsMATE* genes, we identified 11 tandem-duplicated *OsMATE* genes, comprising six gene pairs (Figure S1). Of these, two pairs belonged to Group I and four pairs belonged to Group II (Figure S1). These results indicated that tandem duplication was crucial for the expansion of the *MATE* gene family in *Brachypodium distachyon* and *Oryza sativa*, but had different effects on different *MATE* genomes.

In addition, we found segmental-duplicated gene pairs on the chromosomes of *Brachypodium distachyon* and rice. In *Brachypodium distachyon*, there were five pairs of segmental-duplicated genes identified using DupGen_Finder, with three pairs identified using MCScanX. There were five segmental-duplicated *BdMATE* genes, with one pair in Group I, one pair in Group II, and three pairs in Group IV, respectively. In rice, there are a total of five pairs of segmental-duplicated genes, with one pair in Group I, two pairs in Group II, and two pairs in Group IV. These results show that the *MATE* gene family expanded in both *Brachypodium distachyon* and rice with tandem and segmental duplication as the important driving force.

Then, we evaluated selective pressure exerted on the *MATE* gene family during evolution through Ka (non-synonymous distance), Ks (synonymous distance), and ω (Ka/Ks ratio) values [31]. Based on the neutral theory, it was known that ω values below one indicate purifying selection, while values around one represent neutral evolution and ω values above one indicate positive selection [32].

**Figure 4:**
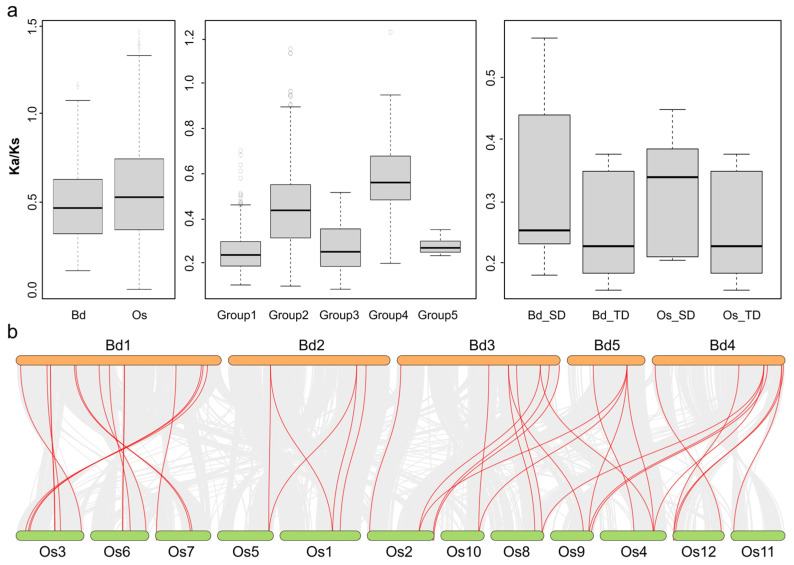
Selection pressure and synteny analyses of *MATE* genes between *Brachypodium distachyon* and *Oryza sativa*. (**a**) Selection pressure between species and groups. (**b**) Collinearity analysis of *MATE* genes. The chromosomes of two Poaceae species are indicated as different colored boxes, respectively. The orange ones on the top are from *Brachypodium distachyon*, with green on the bottom representing *Oryza sativa*. Putative orthologous genes in their genomes are connected by lines using the MCScanX software. Innermost gray solid lines show collinear relationships between *MATE* genes. In total, 40 orthologous *MATE* gene pairs were identified, with red solid lines connected.

The ω values of the *MATE* genes of *Brachypodium distachyon* and *Oryza sativa* were calculated to be less than one, indicating that both *BdMATE* and *OsMATE* genes experienced purifying selection, and the stringency of their selection was basically the same. The ω values within the five groups were calculated to be 0.254, 0.445, 0.268, 0.584, and 0.279, with the smallest value in Group I and the largest value in Group IV, indicating that more stringent purifying selection was carried out within Group I. In addition, we also calculated the ω values of tandem-duplicated and segmental-duplicated genes in *BdMATE* and *OsMATE*, respectively, and found that the ω values of segmental-duplicated genes in *Brachypodium distachyon* and *Oryza sativa* were smaller than those of tandem-duplicated, and were all less than 1 (Figure 4a).

### 2.4. Three-Dimensional Structure Prediction of BdMATEs

**Figure 5:**
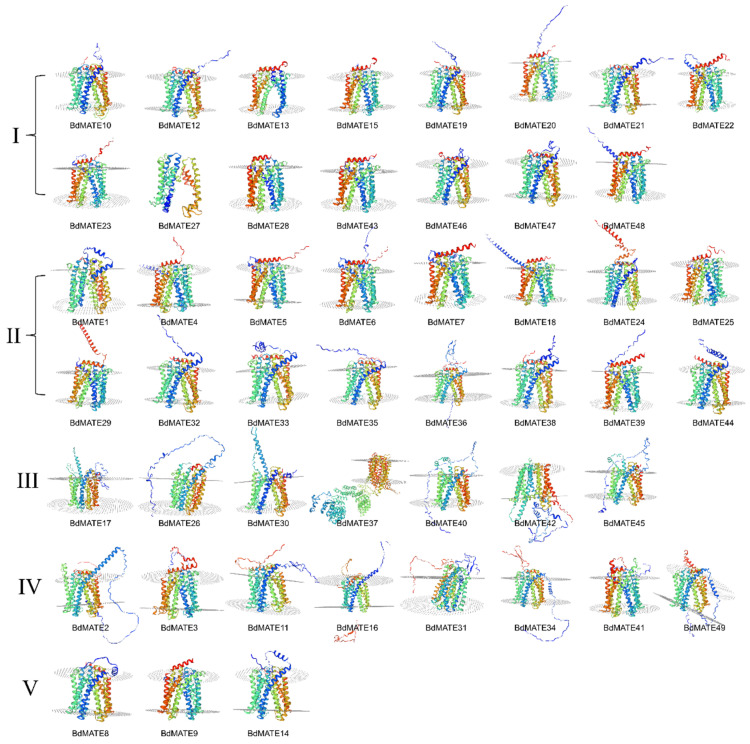
The predicted structure of 49 BdMATE. The BdMATE protein sequences were subjected to the website https://swissmodel.expasy.org (accessed on 15 June 2024). The results showed that the predicted *BdMATE* structure was similar. The protein structure is colored using a rainbow gradient from the N terminus (blue) to the C terminus (red).

To explore the protein structure of BdMATEs, we used the online website https://swissmodel.expasy.org/ (accessed on 15 June 2024) to predict the protein structure of 49 BdMATE (Figure 5). Among the 15 BdMATE predicted conformations in Groups III and IV, in addition to BdMATE17 and BdMATE49, there are one or more long irregular curls in the V-shaped structure of the subject. The third group of BdMATE37 has a large subunit in addition to the V-shaped structure, which may be due to its amino acid sequence being twice as long as other BdMATEs. In other groups, including Groups I, II, and VI, in addition to the V-shaped structure of the main body, most BdMATE also have a regular α-helix. Previously, the structure of *MATE* in *Arabidopsis thaliana*, humans, and bacteria has been reported [8,33,34], and these *MATE* only have V-shaped structures. The additional structure of BdMATE may be related to the special functions it performs.

### 2.5. Collinearity Analysis of MATE Genes between Brachypodium distachyon and Oryza sativa 

We used MCScanX [35] and TBtools software v2.119 [36] for collinearity analysis to allocate orthologous gene pairs between *Brachypodium distachyon* and *Oryza sativa*. We identified 40 putative orthologous *MATE* gene pairs between *Brachypodium distachyon* and *Oryza sativa* genome (Figure 4b). We detected 30 *BdMATE* genes on all five chromosomes of the *Brachypodium distachyon* genome, which formed pairs with 31 *OsMATE* genes mapping to all 12 chromosomes of the *Oryza sativa* genome. Notably, each chromosome of *Brachypodium distachyon* has *MATE* genes that are collinear with rice, and there are 12, 5, 9, 9, and 5 collinear genes on the five chromosomes, respectively (Figure 4b).

### 2.6. Analysis of cis-Regulatory Elements in MATE Promoters 

**Figure 6:**
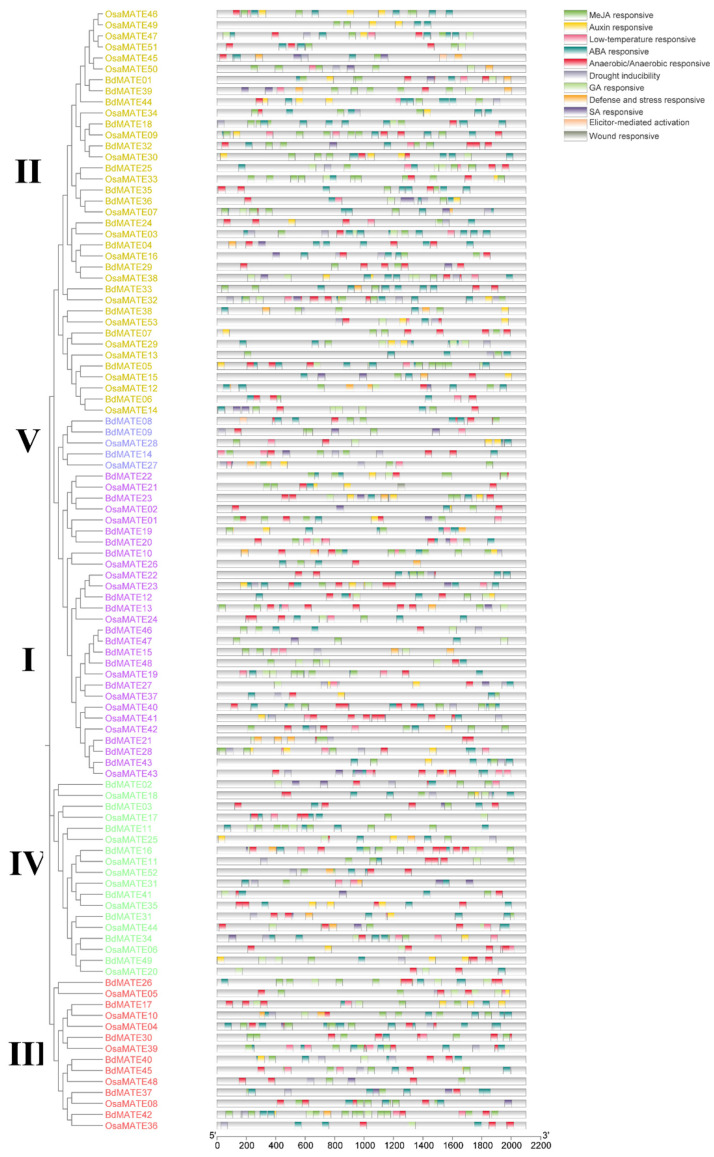
Predictive analysis of cis-regulatory elements in the promoter sequences of *Brachypodium distachyon* and *Oryza sativa MATE* genes. Left: phylogenetic tree of the *B. distachyon* and rice *MATEs* family, replotted from Figure 1. Right: the cis-regulatory elements (CREs) in the 2000 bp upstream regions of the 49 *BdMATEs* and 53 *OsMATEs* genes were predicted using the PlantCare database. These CREs can be divided into two types: phytohormone, including ABA-responsive, Auxin-responsive, GA-responsive, MeJA-responsive, and SA-responsive, and stress, including drought inducibility, low-temperature responsive, elicitor-mediated activation, defense- and stress-responsive, and wound-responsive. Letters I-V present Group I-V.

It has been reported that the *MATE* gene is involved in plant growth and development and defense response to the outside world [2,4,28]. In order to explore the potential functions of *MATE* genes in plant development and under adverse stress, we predicted the 2 kb sequence upstream of *BdMATE* and *OsMATE* genes through the PlantCARE website and analyzed it to predict *cis*-regulatory elements (CREs) in this 2 kb sequence. This analysis identified 11 distinct CREs in the *MATE* promoters, including five phytohormone-responsive CREs and six plant defense response-related CREs (Figure 6). The number of CREs was quite variable across the *B. distachyon* and *Oryza sativa MATE* genes, with the highest number seen in the *B. distachyon BdMATE42* (34 CREs) and the *Oryza sativa OsaMATE04* (36 CREs) promoters, but only 6 CREs in the *OsMATE20* promoter (Figure 6). We identified a total of 1619 potential CREs in *BdMATE* and *OsaMATE* promoters, including 767 CREs in the *BdMATE* promoters. Among the *BdMATE* promoters, we identified 558 elements related to phytohormone responses, which consisted of 173 abscisic acid (ABA)-responsive elements in 43 *BdMATE* promoters, 36 Auxin-responsive elements in 23 *BdMATE* promoters, 44 gibberellic acid (GA)-responsive elements in 29 *BdMATE* promoters, 280 methyl jasmonate (MeJA)-responsive elements in 47 *BdMATE* promoters, and 25 salicylic acid (SA)-responsive elements in 20 *BdMATE* promoters.

Another 209 CREs are related to plant defense responses, including 118 ARE and GC-motifs in 43 *BdMATE* promoters, which mediate anaerobic or anoxic response, 30 drought-inducible sites (MYB-binding site or MBS) in 23 *BdMATE* promoters, 37 low-temperature-responsive elements (LTREs) in 26 *BdMATE* promoters, 23 WUN motifs (AAATTTCCT) in 15 *BdMATE* promoters, responsible for wound-responsive expression, and one AT-rich motif (TAAAATACT), which is responsible for elicitor-mediated activation in the *BdMATE08* promoter.

The above results strongly suggested that *BdMATE* genes participate in plant responses to multiple stresses. A similar analysis identified 852 potential CREs in the promoters of the *OsMATE* genes, including 632 elements related to phytohormone responses and 220 elements related to plant defense responses (Figure 6), underscoring the similar distribution of CREs between *Brachypodium distachyon* and *Oryza sativa MATE* genes. The above results indicate that many *Brachypodium distachyon* and *Oryza sativa MATE* genes may be crucial in plant response to environmental stress because of the various CREs related to phytohormones and plant defense in their promoters.

### 2.7. Analysis of BdMATE Gene Expression Patterns

**Figure 7:**
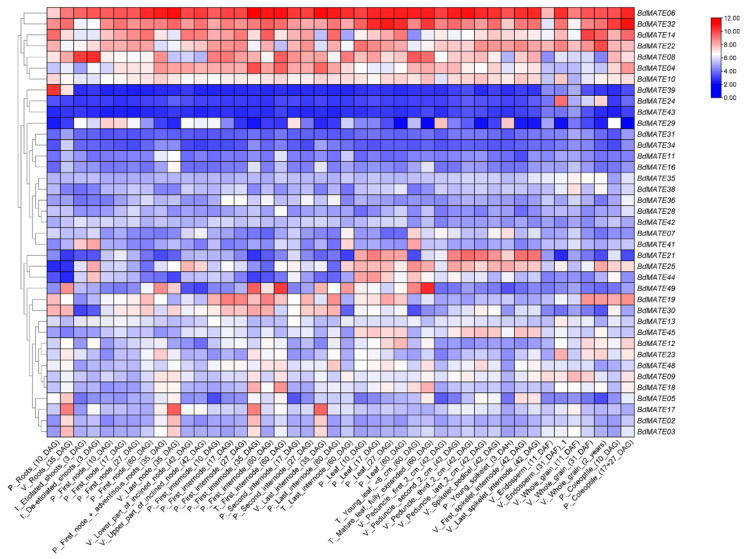
Expression analysis of 49 *BdMATEs*. Expression profiles of 49 *BdMATEs* in plant development. *MATE* genes tissue-specific expression data in *Brachypodium distachyon* were obtained from a published database (Brachypodium eFP Browser (https://bar.utoronto.ca/efp_brachypodium/cgi-bin/efpWeb.cgi accessed on 10 September 2023)). The color bar represents the expression value, shown on the right side of the map. The heat map with phylogenetic tree was drawn with Tbtools.

To assess the role of *Brachypodium distachyon MATE* genes in plant development, we turned to transcriptome deep-sequencing (RNA-seq) datasets from the Brachypodium eFP Browser database [37], which we collected and analyzed as previously described [38]. We compiled the expression profiles of all *BdMATE* genes across 44 different tissues and organs and standardized these data through the Z-score. If the Z-score is positive, this means that the data are greater than the average value; if the Z-score is negative, this means that the data are less than the average value. We then visualized transcript levels as a heat map, which illustrated the differences in expression patterns observed for *BdMATE* genes, and used the clustering function of TBtools for ordering the genes (Figure 7). According to the criterion of in the monocots, we have screened 12 highly expressed *BdMATE* genes (Z-score greater than four) from 39 *BdMATE* genes. Interestingly, we observed that these highly expressed genes are generally expressed in different *Brachypodium distachyon* tissues, and most of the highly expressed genes were expressed in vegetative tissues (Figure 7). Within the same group, *BdMATE* genes showed distinct expression profiles in different tissues (Figure 7), suggesting sub-functionalization or functional diversification.

Among the *BdMATE* genes in Group I, three high-expression genes were detected, of which *BdMATE22/43* was highly expressed in wholegrain and *BdMATE28* was highly expressed in internode. Four high-expression genes were detected in Group II, *BdMATE18/24/38/39* was highly expressed in internode, endosperm, wholegrain, and roots, respectively, five high-expression genes were found in Group IV, *BdMATE03/34* was highly expressed in roots, *BdMATE11/16* was highly expressed in nodes, and *BdMATE49* was highly expressed in mature leaves. However, no high-expression genes were found in Group III and Group V. This shows that it has the function of tissue- or organ-specific regulation. Further investigation indicated that tandem-duplicated *BdMATE* genes were differentially expressed in the selected samples (Figure 7), suggesting their functional differentiation.

### 2.8. Expression Analysis of Phytohormone-Treated BdMATE Genes

**Figure 8:**
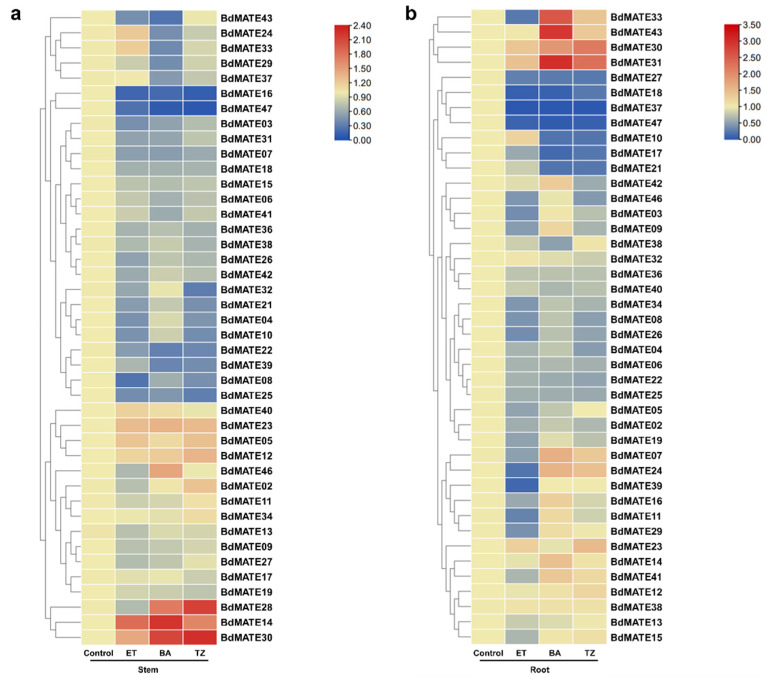
Expression of *BdMATE* genes after hormone treatment. qRT-PCR analysis of 43 *BdMATE* genes. (**a**) Expression of *BdMATE* genes in leaves after three hormone treatments. (**b**) Expression of *BdMATE* genes in roots after three hormone treatments. *BdMATE* transcript levels were normalized using *BdUBI-18* as the internal reference. Each data point represents the average of three biological repeats.

Hormones are essential for plant growth, development, and stress resistance. In order to identify the effects of plant hormones on *BdMATE* expression, we performed RT-qPCR analysis on *BdMATE* treated with three hormones: ET, 6-BA, and tZ. The results are presented as a heat map (Figure 8 and Figure S2). In the above-ground part, after three hormone treatments, the expression of *BdMATE14* was all up-regulated, and the expression of *BdMATE16/22/25/49* was all down-regulated. The expression of *BdMATE03/07/33/45* was down-regulated after ET and 6-BA treatment, but there was no significant change in expression after TZ treatment. The expression of *BdMATE04/08/10/22/34* was down-regulated after 6-BA and TZ treatment, but there was no significant change in expression after ET treatment. The expression of *BdMATE4* was down-regulated after ET and TZ treatment, but there was no significant change in the expression after the 6-BA treatment. The expression levels of *BdMATE24/31/35/39* all decreased after 6-BA treatment, while the remaining two hormones showed no significant changes after treatment.

In roots, the expression of *BdMATE32/33* was up-regulated after 6-BA and TZ treatment, but there was no significant change in expression after ET treatment. The expression level of *BdMATE07/35* was down-regulated after ET treatment, up-regulated after 6-BA treatment, and had no significant change after TZ treatment. After three hormone treatments, the expression of *BdMATE17/18/29/39/49* decreased. The expression of *BdMATE08/28/48* was down-regulated after ET and TZ treatment, but there was no significant change in expression after 6-BA treatment. The expression of *BdMATE10/21/22* was down-regulated after 6-BA and TZ treatment, but there was no significant change in expression after ET treatment. The expression levels of *BdMATE03/05/11/16/19/24/31/36/41* were all down-regulated after ET treatment, and there was no significant change in expression levels after 6-BA and TZ treatment. The expression levels of *BdMATE04/25/44* were all down-regulated after TZ treatment, and there was no significant change in expression levels after ET and 6-BA treatments.

### 2.9. BdMATE Genes Subcellular Location

**Figure 9:**
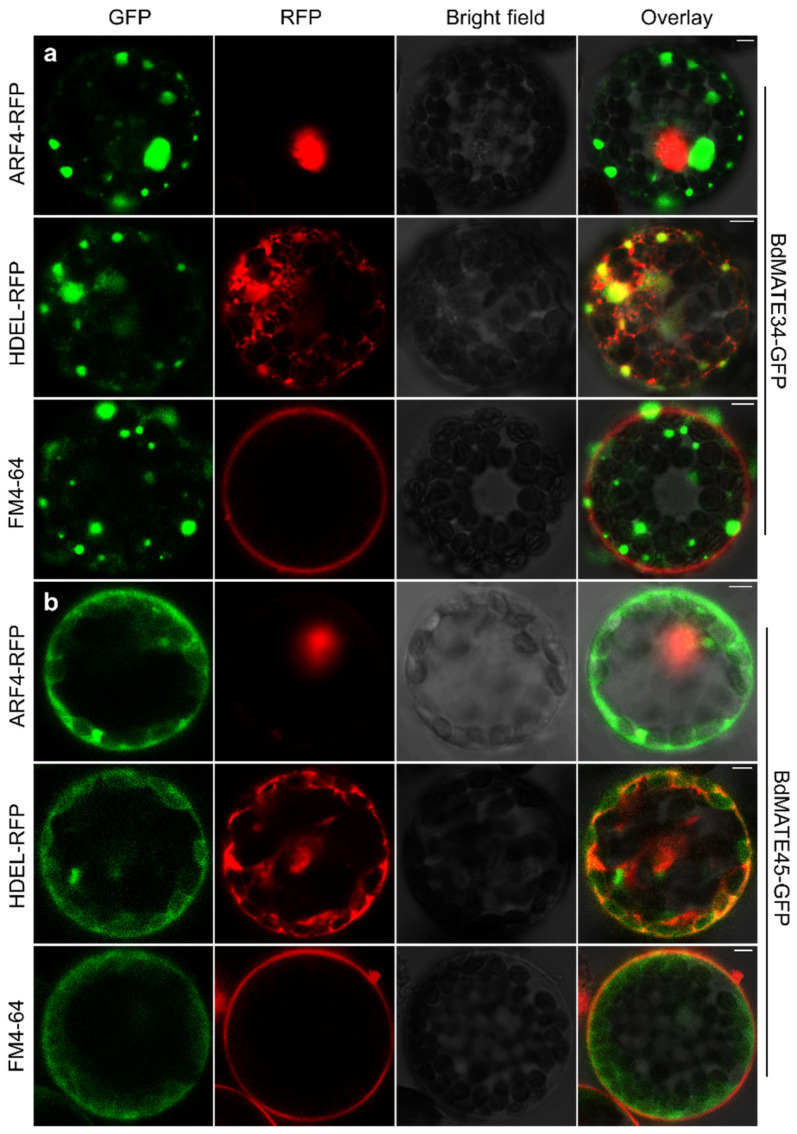
Subcellular localization of BdMATE34 and BdMATE45. BdMATE34 belongs to Group IV, and BdMATE40 belongs to Group III. ARF4 is the marker of nucleus, HDEL is the marker of endoplasmic reticulum, and FM4-64 is the marker of cell membrane. (**a**) Subcellular localization of BdMATE34. (**b**) Subcellular localization of BdMATE45. Scale bars, 5 μm.

In order to determine the subcellular location of *BdMATE*, we selected *BdMATE34* and *BdMATE45* for experiments. Subcellular localization results showed that BdMATE34 was fluorescent on the cell membrane and blocked fluorescence in the cytoplasm. There is no co-localization with the cell nuclear marker and the cell membrane marker FM4-64, but there is co-localization with the endoplasmic reticulum marker HDEL. BdMATE45 has a different subcellular localization, and its fluorescence is mainly in the cell membrane and cytoplasm (Figure 9). There is no co-localization with the cell nuclear marker, but there is co-localization with the cell membrane marker FM4-64, the endoplasmic reticulum marker HDEL, and the endoplasmic reticulum–plasma membrane junction marker. In addition, BdMATE34 belongs to Group IV, and BdMATE45 belongs to Group III. BdMATE34 is mainly distributed in blocks in the cytoplasm, but this phenomenon is not seen in the localization of BdMATE45 (Figure 9). The different localizations between subfamily groups suggest that BdMATE has undergone functional differentiation during evolution.

## 3. Discussion

In plants, MATE proteins have a wide range of functions and are involved in the transport of foreign substances and their own metabolites, immune responses, etc. [39,40]. Currently, MATEs have been widely identified and evolutionarily analyzed in plants [9,16,18,19,20,21,22,23,24,28,29,41,42,43,44,45,46,47,48,49]. *MATE* genes in *Brachypodium distachyon* were first identified by R. Contreras, with some phylogenetic tree analysis [50]. Here, we conducted a detailed genome-wide analysis and identification of *BdMATE* and identified a total of 49 *BdMATE* genes. Subsequently, by analyzing the phylogeny, gene structure, and expression patterns of BdMATE, we revealed the evolution and potential functions of *MATE* genes, which will help us to understand the functions of MATE transporters in Poaceae.

### 3.1. MATE Gene Family Conservation in the Poaceae 

There were only two *MATE* genes identified in the human genome [51,52], compared with 49 *MATE* genes revealed in *Brachypodium distachyon* in our study (Table S1). The *MATE* gene family has greatly expanded in plants relative to other kingdoms [9,53], suggesting their diverse and vital roles in plants. The amino acid composition of BdMATE proteins ranged from 332 to 606 amino acids, except for BdMATE37, which consisted of 1403 amino acids. Our analysis predicted that most MATE proteins localize to the plasma membrane, which would be consistent with their roles as transporters of toxic compounds [8], thereby conferring resistance to the toxin. A phylogenetic tree was constructed using 49 *BdMATEs*, 53 *OsMATEs*, 56 *AtMATEs*, and other 30 functional published *MATE* genes (Figure 1), which we validated based on their gene structures and the organization of their encoded functional motifs (Figure 2). In agreement with the phylogenetic analysis, gene structures, the number of exons, the number of TM domains, and the predicted subcellular locations showed higher similarity within each group than between groups, supporting our classification of *MATE* members. *MATE* family members from Group III only displayed two to five conserved motifs, but had the most exons relative to all other groups (Figure 2), indicating large structural differences in the *MATE* genes and variation in the function of the encoded proteins.

### 3.2. Tandem Duplications Contributed to MATE Gene Expansion in Brachypodium distachyon

The distribution of *Brachypodium distachyon MATE* genes across their genomic chromosomes was uneven, a pattern previously noted in the tomato *MATE* family [2] that indicated the occurrence of an aneuploidy event [54,55]. Intra- and inter-synteny and collinearity analysis suggested that *BdMATE* genes may have expanded by tandem duplication, as in tomato [2], resulting in the tight linkage of *MATE* genes in clusters in the Poaceae (Figure 3 and Figure S1), and implying that tandem duplications may have contributed to the expansion of the *MATE* gene family in the Poaceae. The ω values of *Brachypodium distachyon* and rice are 0.493 and 0.562, respectively. Importantly, these values were less than one, indicating purifying selection during the evolution of the *MATE* gene family in *Brachypodium distachyon*. We found three pairs of segmental duplication *MATE* genes in Groups I, III, and IV of *Brachypodium distachyon*, and five pairs of segmental duplication genes in Groups I, II, and IV of rice, suggesting that the expansion of the *MATE* gene family may be driven by different mechanisms between Poaceae. In addition, we also found different numbers of tandem duplications of *MATE* genes in *Brachypodium distachyon* and rice. We found tandem duplication genes in all five groups of *Brachypodium distachyon* for a total of eight pairs. In rice, tandem duplication genes were only found in Groups I, II, and IV, for a total of eight pairs. These results suggest that diversification has occurred in different groups of *Brachypodium distachyon* and rice, especially in genes belonging to Groups I and IV.

### 3.3. MATE Genes Function and Gene Expression

Understanding the changes in gene expression when plants respond to stress can not only be used to evaluate their gene functions, but also increase our understanding of the stress resistance mechanisms of plants in nature [56]. Therefore, we analyzed the expression level of *BdMATE* after *Brachypodium distachyon* was treated with different hormones to predict its function [38]. *MATEs* are relatively abundant in plants and are involved in the transport of secondary metabolites, the transport of plant hormones, and the maintenance of metal ion homeostasis [4]. An analysis of RNA-seq data across 44 tissues or organs from the Brachypodium eFP Browser database established that many *BdMATE* genes were preferentially expressed in vegetative tissues, including root, node, and leaf (Figure 6), indicating that these genes may be related to material transport. The fourth group of *BdMATE* genes had the most highly expressed genes, and all five of these *BdMATE* genes were highly expressed in vegetative tissues. *BdMATE39* from the second group had a positive Z Score only in the root and was highly expressed in R10, while it was negative in the rest of the tissues or organs, indicating that *BdMATE39* may be specifically involved in the root, especially in the R10 stage.

We also screened the cis-regulatory elements of all *Brachypodium distachyon BdMATE* gene promoters and identified 767 CREs, including 280 MeJA-responsive CREs, 173 ABA-responsive CREs, 118 anaerobic/anaerobic-responsive CREs, 44 GA-responsive CREs, 37 low-temperature-responsive CREs, 36 Auxin-responsive CREs, 30 drought-inducible CREs, 23 defense- and stress-responsive CREs, and one elicitor-mediated activation CREs (Figure 5). We identified more CREs in rice *OsMATE*, including 264 MeJA-responsive, 252 ABA-responsive, 123 anaerobic/anaerobic-responsive, 45 Auxin-responsive, 44 GA-responsive, 43 drought-inducible, 34 low-temperature-responsive, 27 SA-responsive, 18 defense- and stress-responsive, one wound-responsive, and one elicitor-mediated activation. This suggests that *BdMATE* and *OsMATE* genes may play an important role in plant development and adaptation to environmental conditions.

Due to the influence of various environmental pressures on plants during their growth process, they have evolved multiple response mechanisms to cope with the pressures brought about by environmental changes [57]. MATE is a transporter protein that could increase plant stress resistance by exporting toxic substances, such as heavy metals, metabolites, phylogenetic topology among *Brachypodium distachyon*, and other experimentally identified *MATE* genes could be used to predict their similar gene functions [2].

Phylogenetic analysis revealed that Group I contained 15 *BdMATEs*, 13 *OsMATEs*, and 17 *AtMATEs* (Figure 1). Notably, the transport function of specific *MATE* genes, such as *NtJAT1* and *AtDTX1*, has been established for their role in alkaloid translocation from cytosol to the vacuole, which modulates plant development and confers resistance to diseases [58,59,60], suggesting that *MATE* genes in Group I could be integral to the alkaloid transport mechanism and plant resistance to disease. Group II encompassed 16 *BdMATEs*, 21 *OsMATEs*, and 22 *AtMATEs*, the biggest subfamily (Figure 1). A subset of *MATE* genes in Group II is implicated in the transport of secondary metabolites, including proanthocyanin, flavonoids, nicotine, and sugar. GFD1 has been shown to interact with the sugar transporters OsSWEET4 and OsSUT2, thereby regulating the allocation of starch within grains and stems and impacting the overall carbohydrate distribution within the plant [14]. In Arabidopsis, TT12/DTX41 is known to transport anthocyanin [61] and epicatechin 3′-O-glucoside [62], and is implicated in the vacuolar sequestration of flavonoids [63], similar to DTX35/FFT [64]. The homologous gene *BrTT12* from rapeseed also contributes to seed coat pigmentation [65]. In grapevine, VvAM1 and VvAM3 are experimentally proven to transport anthocyanins [66,67]. In tobacco, NtMATE21 and NtMATE22 are associated with flavonol transport, which affects plant growth and development [13]. Although the functions of BdMATEs have not been characterized, their sequence similarity to MATEs from other species hints at a potential role for their function in mediating the transport of secondary metabolites. Group III included a modest collection of seven *BdMATEs*, seven *OsMATEs*, and six *AtMATEs*. Three *OsMATE* genes, *OsFRDL1*/*OsMATE10* [68,69,70], *OsFRDL4*/*OsMATE4* [71,72], and *OsFRDL2*/*OsMATE40* [69], and two *AtMATE* genes, *AtMATE* [73,74] and *AtFRD3* [75], were implicated in processes of transporting aluminum or iron for detoxification or translocation. According to the sequence similarity, these findings suggested that *BdMATE* genes in Group III were strong contenders for roles in aluminum detoxification and iron translocation mechanisms. Group IV encompassed eight *BdMATEs*, ten *OsMATEs*, and nine *AtMATEs*. In Arabidopsis, BCD1, also known as ZRZ or ABS4, has been implicated in iron homeostasis and hypocotyl cell elongation [76,77,78]. Additionally, ELS1, also named ABS3L1, and DTX50, known as ABS3L2, have been demonstrated to play a role in modulating cell elongation [79,80]. Conversely, ADS1, also referred to as ABS3 or ADP1, is reported to exert a negative regulatory effect on hypocotyl cell elongation and plant disease resistance [78,81,82,83,84]. The Arabidopsis gene *DTX18* has been characterized for its role in exporting hydroxycinnamic acid amides to the leaf surface, which serves to inhibit the germination of *Phytophthora infestans* spores [85]. Furthermore, ABERRANT LATERAL ROOT FORMATION 5 (ALF5) has been identified as an efflux transporter, which is crucial for root detoxification [86]. The above results provide valuable information for further functional characterization of *MATE* genes during development and under stress conditions. 

In order to analyze the differences in the functions of different BdMATE subfamilies, we observed the subcellular localization of BdMATE34 from Group IV and BdMATE45 from Group III. The fluorescence of BdMATE34 is mainly localized in the cytoplasm, showing an irregular block structure, and its fluorescence signal is not present on the cell membrane. The fluorescence of BdMATE45 is mainly concentrated in the cell membrane and the nearby cytoplasm. In addition, neither of these two proteins is localized in the nucleus. These two distinct subcellular localizations may be related to the fact that BdMATE34 and BdMATE45 belong to different subfamilies and perform different functions in cells.

## 4. Materials and Methods 

### 4.1. MATE Gene Identification

We used Arabidopsis MATE proteins [28] as query words and searched for MATE proteins of Bd using the Basic Local Alignment Search Tool for Proteins (BLASTP) to identify the family members of *MATE* genes in *Brachypodium distachyon*. In addition, we also searched for candidate genes of BdMATE in the Ensembl database [87] using the Pfam entry PF01554 of the MATE domain downloaded from the Pfam database [88] using HMMER3.0 software [89] with an E-value cutoff of 10^−5^, as previously described [90]. Next, we performed protein scanning using the SMART [91], CDD [92], InterProscan [93], and Pfam [88] databases to further confirm whether the candidate MATE proteins have complete domains.

### 4.2. Analysis of Gene Structure and Domain Architecture 

Using the Gene Structure Display Server (GSDS 2.0), accessible at http://gsds.cbi.pku.edu.cn (accessed on 9 September 2023) [94], coupled with TBtools [36], the gene exon–intron structure of the *MATE* genes was delineated. The amino acid sequences of these MATE proteins were subjected to a comprehensive search across the SMART database [91], InterProscan [93], and MEME suite [95] to characterize the associated functional domains.

### 4.3. Prediction of cis-Regulatory Elements in Gene Promoters and Subcellular Localization of Proteins

The 2 kb sequence upstream of the transcription start site for each MATE gene promoter was extracted. The PlantCARE database, available at website (http://bioinformatics.psb.ugent.be/webtools/plantcare/html) [96] was utilized to identify potential cis-regulatory elements for in-depth analysis. Concurrently, the subcellular localization of the MATE proteins was ascertained through the WoLF PSORT database [97], available at https://psort.hgc.jp.

### 4.4. Phylogenetic Analysis of MATE Genes

The sequence alignment of all MATE domain-containing proteins was performed, with the MUSCLE algorithm. Subsequently, a phylogenetic tree was constructed employing the maximum likelihood approach in MEGA X [98]. The optimal amino acid substitution model, LG + G + F, was selected, with pairwise deletion of gaps and 1000 bootstrap replicates. The resultant phylogenetic topology tree was graphically represented using FigTree software.

### 4.5. Analysis of Gene Duplication and Synteny 

The intragenomic synteny and intergenomic collinearity blocks within the genome of *Brachypodium distachyon* were conducted with the MCScanX software [35], which was executed with its standard parameter settings. In addition, the duplicate genes were also identified by DupGen_Finder (https://github.com/qiao-xin/DupGen_finder, accessed on 14 August 2024) [30], and *Spirodela polyrhiza* was used as an outgroup. The union of genes identified by the above two methods was considered a duplication classification. The detection of both tandem and segmental duplications was carried out according to the established methods outlined in previous studies [99], which was visualized by Tbtools software [36].

### 4.6. Gene Expression Profiling of MATE Genes in Brachypodium distachyon

The tissue-specific expression profiles of *MATE* genes in *Brachypodium distachyon* were sourced from the RNA-seq data available in the Brachypodium eFP Browser (https://bar.utoronto.ca/efp_brachypodium/cgi-bin/efpWeb.cgi) [37]. These datasets were analyzed to profile differential gene expression, which was subsequently clustered by employing R 4.2.0 software, following previously established protocols [100,101].

### 4.7. Quantitative RT-PCR Analysis

We used 1/2 Hogaland medium to culture *Brachypodium distachyon*. After treating 4-week-old *Brachypodium distachyon* with 1/2 Hogaland culture medium containing 100 nM trans-Zeatin, 10 µM 6-benzylaminopurine (6-BA) or 3 µL/L ethylene for 24 h, we took out the plants and divided them into seedlings and roots according to the position of the embryo. After rapid freezing in liquid nitrogen, the samples were stored in a refrigerator at −80 °C. We performed qRT-PCR (quantitative reverse transcription PCR) analysis, as previously described [102,103]. After quantifying the expression levels of each gene in various samples and obtaining three replicated Ct values, the Ct difference between the experimental and control groups and the internal reference gene BdUBC18 (ΔCt) should be calculated. Subsequently, the difference in ΔCt between the experimental group and the control group (ΔΔCt) should be determined. Finally, the 2^−ΔΔCt^ method should be employed to assess the relative changes in Ct values of the experimental group compared to the control sample. Hypothesis testing should be conducted using a Student’s *t*-test. Primers for the BdMATE gene were obtained from the qprimerDB database [104] (Table S4).

### 4.8. Subcellular Localization of the BdMATE

To determine the subcellular localization of BdMATE34/40/45/49, we constructed a pUC121-BdMATE34/40/45/49XTEN-GFP-HA vector that can express BdMATE34/40/45/49 fusion protein with a GFP tag. The constructed vector was transformed into Arabidopsis protoplasts for expression and observed using a laser confocal microscope (LSM880), as previously described [105,106].

## 5. Conclusions

The *MATE* gene family of *Brachypodium distachyon* and *Oryza sativa* in Poaceae was comprehensively identified and analyzed. In total, 49 *BdMATE* genes were found in *Brachypodium distachyon* and 53 *OsMATE* genes were found in rice, and they were unevenly distributed on chromosomes. We divided *MATE* genes into five groups through phylogenetic analysis, which was also verified by subsequent gene structure analysis. The ω value indicates that *MATE* genes have been subjected to purifying selection during evolution, and segmental duplication genes are the main driving force. Analysis of cis-regulatory elements and expression patterns of *MATE* promoters showed that *MATE* genes showed gene diversity under different environmental pressures and different growth and development stages.

## Data Availability

Data are contained within the article and Supplementary Materials.

## Supplementary Materials

The following supporting information can be downloaded at: https://www.mdpi.com/article/10.3390/plants13182586/s1, Figure S1. Chromosomal distribution of *Oryza sativa MATE* genes. The number of chromosomes is indicated on the left of each chromosome (vertical bar). The size of chromosomes is indicated by its relative length by using the information from Ensemble database. Tandem duplicated genes are connected in red arc lines. Figure S2. Expression of *BdMATE* genes after hormone treatment. qRT-PCR analysis of 43 *BdMATE* genes. (a) Expression of *BdMATE* genes in leaves after three hormone treatments. (b) Expression of *BdMATE* genes in roots after three hormone treatments. *BdMATE* transcript levels were normalized using *BdUBI-18* as the internal reference. Each data point represents the average of three biological repeats. Statistical significance was determined using Student’s *t*-test, with * *p* < 0.05 and ** *p* < 0.01 indicating significant differences. Table S1. The evolutionary relationship and physical properties of BdMATE; Table S2. Subcellular prediction results of BdMATE; Table S3. List of *BdMATE* duplication genes identified by DupGen_Finder; Table S4. List of *BdMATE* genes primers.

## Abbreviations

ABA	abscisic acid
Bd	*Brachypodium distachyon*
CRE	*cis*-regulatory element
GA	gibberellin
GSDS	Gene Structure Display Server
HMM	hidden Markov model
IAA	indole-3-acetic acid
Ka	non-synonymous distance
Ks	synonymous distance
MATE	Multidrug and Toxic Compound Extrusion
MeJA	methyl jasmonate acid
MEME	multiple EM for motif elicitation
MUSCLE	Multiple Sequence Comparison by Log Expectation
MW	molecular weight
Os	*Oryza sativa*
pI	isoelectric point
qRT-PCR	quantitative reverse transcription PCR
SA	salicylic acid
