# Supplementary material for: Comprehensive Identification and Expression Analysis of the Multidrug and Toxic Compound Extrusion (MATE) Gene Family in Brachypodium distachyon
Source: Plants (Basel). 2024 Sep 15;13(18):2586. doi: 10.3390/plants13182586 (PMC11434668; doi:10.3390/plants13182586)
Supplement: Supplementary file 1 [file plants-13-02586-s001.zip › plants-3139341-supplementary.pdf]

Supplemental Data

Comprehensive Identification and Expression Analysis of the Multidrug and Toxic Compound Extrusion (MATE) Gene Family in Brachypodium Distachyon

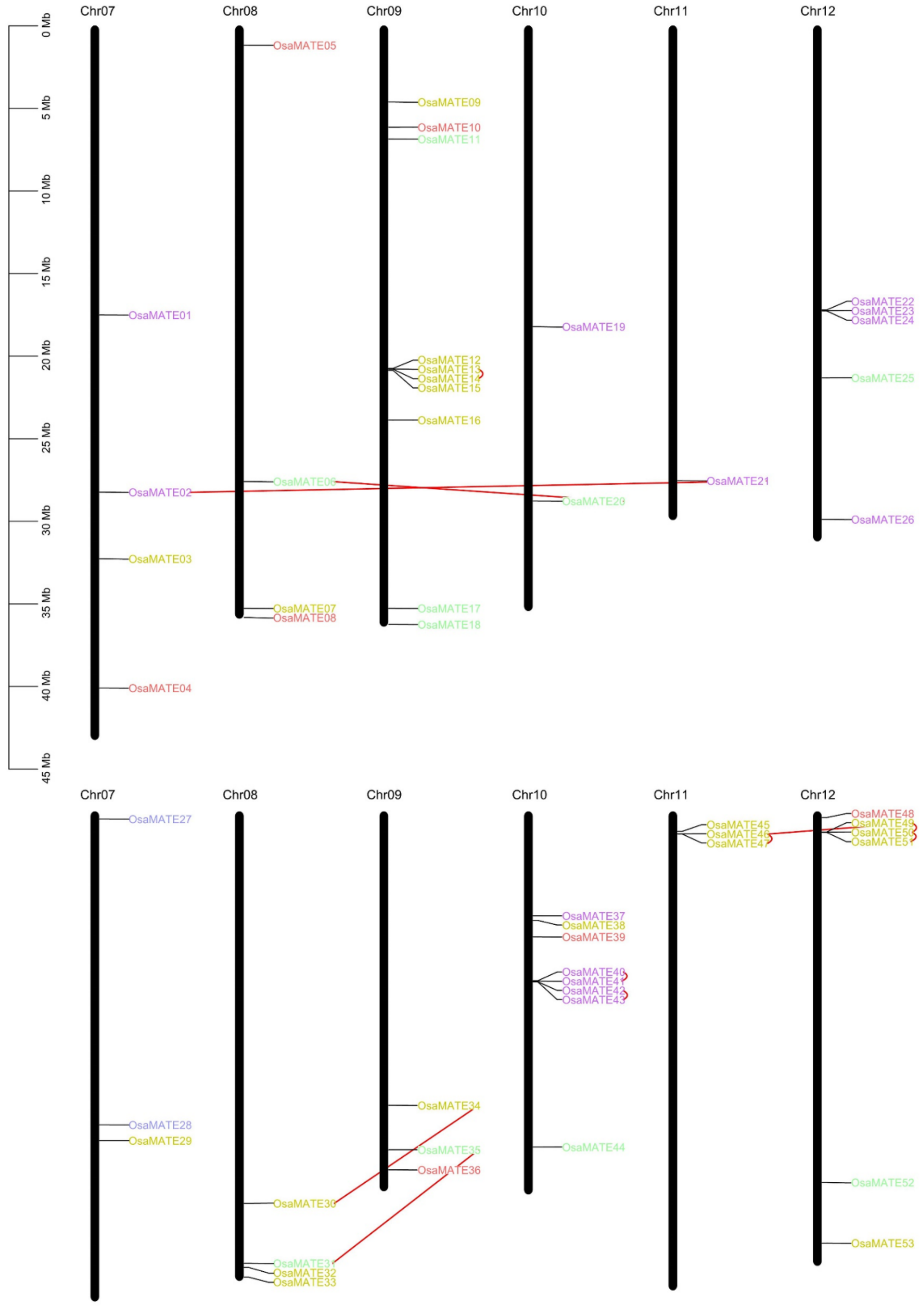

Figure S1. Chromosomal distribution of *Oryza sativa MATE* genes. The number of chromosomes is indicated on the left of each chromosome (vertical bar). The size of chromosomes is indicated by its relative length by using the information from Ensemble database. Tandem duplicated genes are connected in red arc lines.

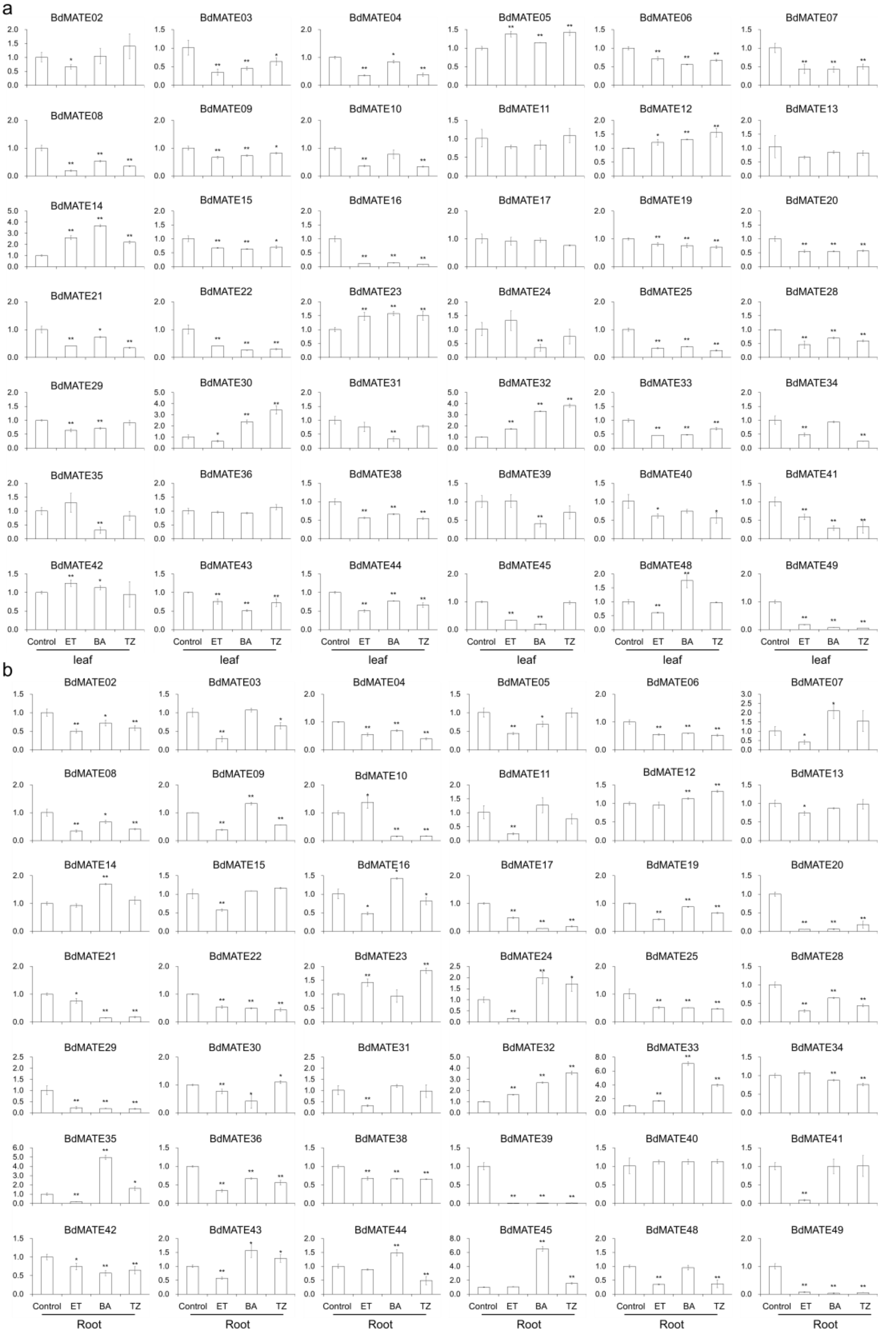

Figure S2. Expression of *BdMATE* genes after hormone treatment. qRT-PCR analysis of 43 *BdMATE* genes. (a) Expression of *BdMATE* genes in leaves after three hormone treatments. (b) Expression of *BdMATE* genes in roots after three hormone treatments. *BdMATE* transcript levels were normalized using *BdUBI-18* as the internal reference. Each data point represents the average of three biological repeats. Statistical significance was determined using Student's t-test, with *P < 0.05 and **P < 0.01 indicating significant differences.

Table S1. The evolutionary relationship and physical properties of BdMATE

Gene name	Sequence ID	Number of Amino Acid	Molecular Weight (KDa)	Theoretical pI	Transmembrane domain count	Phylogenetic tree
BdMATE01	Bradi1g00272.1	449	48.32	7.65	12	II
BdMATE02	Bradi1g00780.2	606	64.39	9.44	8	IV
BdMATE03	Bradi1g02370.1	527	54.50	8.68	12	IV
BdMATE04	Bradi1g14350.1	492	53.17	9.08	12	II
BdMATE05	Bradi1g15670.1	494	53.04	5.5	12	II
BdMATE06	Bradi1g15680.1	512	54.78	6.88	12	II
BdMATE07	Bradi1g26240.1	496	52.38	6.82	12	II
BdMATE08	Bradi1g26800.2	477	51.35	7.01	12	V
BdMATE09	Bradi1g26810.1	487	52.33	7.04	12	V
BdMATE10	Bradi1g34660.2	497	54.28	7.46	8	I
BdMATE11	Bradi1g37947.1	571	59.88	7.78	10	IV
BdMATE12	Bradi1g42390.1	479	51.35	6.7	12	I
BdMATE13	Bradi1g42410.1	396	43.25	8.19	10	I
BdMATE14	Bradi1g59200.1	489	52.74	6.12	11	V
BdMATE15	Bradi1g66270.1	480	51.79	7.57	9	I
BdMATE16	Bradi1g69120.1	619	63.37	7.54	9	IV
BdMATE17	Bradi1g69770.1	559	58.99	6.98	8	III
BdMATE18	Bradi1g72007.1	506	55.17	5.64	10	II
BdMATE19	Bradi2g12080.1	486	52.34	6.02	10	I
BdMATE20	Bradi2g16223.1	455	49.10	6.6	6	I
BdMATE21	Bradi2g16887.2	475	50.77	8.15	10	I
BdMATE22	Bradi2g17260.1	514	54.51	7.55	10	I
BdMATE23	Bradi2g46750.1	492	51.63	8.37	11	I
BdMATE24	Bradi2g50927.2	533	57.63	9.73	11	II
BdMATE25	Bradi2g57310.1	482	51.20	5.14	12	II
BdMATE26	Bradi3g02170.5	546	58.97	8.79	9	III
BdMATE27	Bradi3g11205.2	332	36.01	10.04	5	I
BdMATE28	Bradi3g22880.2	472	50.42	8.45	11	I
BdMATE29	Bradi3g23000.4	499	53.71	8.59	10	II
BdMATE30	Bradi3g23150.1	502	53.70	8.55	9	III
BdMATE31	Bradi3g31580.1	562	57.79	6.52	10	IV
BdMATE32	Bradi3g38450.1	489	52.90	8.15	10	II
BdMATE33	Bradi3g42290.1	510	54.38	5.99	12	II
BdMATE34	Bradi3g51590.1	582	60.95	7.9	12	IV
BdMATE35	Bradi3g55790.1	514	53.89	5.37	12	II
BdMATE36	Bradi3g55800.2	571	60.67	5.62	10	II
BdMATE37	Bradi3g60830.2	1403	150.32	6.91	9	III
BdMATE38	Bradi4g01610.1	504	54.66	6.33	11	II
BdMATE39	Bradi4g12390.1	496	53.73	8.75	12	II
BdMATE40	Bradi4g26770.1	552	57.45	5.89	9	III

BdMATE41	Bradi4g35660.1	524	54.88	8.41	11	IV
BdMATE42	Bradi4g37097.1	571	60.69	9.62	10	III
BdMATE43	Bradi4g39390.3	474	50.53	8.52	12	I
BdMATE44	Bradi4g43900.1	506	55.45	6.61	10	II
BdMATE45	Bradi4g44826.1	550	57.89	6.38	10	III
BdMATE46	Bradi5g07146.2	467	50.48	8.11	7	I
BdMATE47	Bradi5g07152.1	480	51.73	8.79	12	I
BdMATE48	Bradi5g07160.1	481	51.84	8.65	12	I
BdMATE49	Bradi5g18830.1	580	60.81	8.43	12	IV

Table S2. Subcellular prediction results of BdMATE

Sequence ID	Plasma Membrane	Vacuole	ER.	golgi	Chloro -plast	Extracellular	Nucleus	Cytopla -sm	peroxisome
BdMATE01	12	2							
BdMATE02	12		2						
BdMATE03	11	1	1	1					
BdMATE04	7		5	2					
BdMATE05	4	8		2					
BdMATE06	6	5		3					
BdMATE07	9	3		2					
BdMATE08	10	2		2					
BdMATE09	8	3	1	2					
BdMATE10	7		5	2					
BdMATE11	11	1	1		1				
BdMATE12	9	2	1	2					
BdMATE13						2	12		
BdMATE14	10	2	1				1		
BdMATE15						2	12		
BdMATE16	8	2	2	2					
BdMATE17	3	8		2				1	
BdMATE18	11		1	2					
BdMATE19	8	4		2					
BdMATE20	7	5	1	1					
BdMATE21						2	12		
BdMATE22	6	5		2				1	
BdMATE23	3	2	6	2		1			
BdMATE24						2	12		
BdMATE25	9	5							
BdMATE26	8		1		5				
BdMATE27	5	5	2			2			
BdMATE28	9	2	2				1		
BdMATE29	9		5						
BdMATE30	9	2	1	1				1	
BdMATE31	9	2	1	2					
BdMATE32	11	2	1						
BdMATE33	10	3	1						
BdMATE34	8	2	3				1		
BdMATE35	10	1	2		1				
BdMATE36	8	1	3		2				
BdMATE37	9	1	2		1		1		
BdMATE38	6	4	1	3					
BdMATE39	13	1							
BdMATE40	4	1	1		7				1

BdMATE41	7	3	3		1			
BdMATE42	12	1	1					
BdMATE43						2	12	
BdMATE44	12	2						
BdMATE45	7		1		6			
BdMATE46						2	12	
BdMATE47						2	12	
BdMATE48	6	4	2	2				
BdMATE49	8	3		2				1

Table S3. List of *BdMATE* duplication genes identified by DupGen_Finder.

Gene name	
**Whole-genome duplication genes**	**5 pairs of genes**
Bradi2g17260	Bradi2g46750
Bradi3g31580	Bradi5g18830
Bradi3g51590	Bradi5g18830
Bradi4g26770	Bradi4g44826
Bradi4g35660	Bradi5g18830
**Tandem duplication genes**	**3 pairs of genes**
Bradi1g26800	Bradi1g26810
Bradi3g55790	Bradi3g55800
Bradi5g07146	Bradi5g07149
**Proximal duplication genes**	**3 pairs of genes**
Bradi1g15670	Bradi1g15680
Bradi1g42390	Bradi1g42410
Bradi5g07152	Bradi5g07160
**Transposed duplication genes**	**12 pairs of genes**
Bradi1g00780	Bradi3g31580
Bradi1g02370	Bradi5g18830
Bradi1g37947	Bradi3g31580
Bradi1g69120	Bradi3g51590
Bradi1g69770	Bradi4g44826
Bradi2g12080	Bradi2g46750
Bradi2g12080	Bradi2g17260
Bradi2g16223	Bradi2g46750
Bradi3g02170	Bradi4g26770
Bradi3g23150	Bradi4g26770
Bradi3g60830	Bradi4g26770
Bradi4g37097	Bradi4g44826
**Dispersed duplication genes**	**41 pairs of genes**
Bradi1g00272	Bradi4g12390
Bradi1g00780	Bradi4g35660
Bradi1g02370	Bradi3g51590
Bradi1g14350	Bradi3g23000
Bradi1g15670	Bradi1g26240
Bradi1g15680	Bradi1g26240
Bradi1g26240	Bradi4g01610
Bradi1g26800	Bradi1g59200
Bradi1g26810	Bradi1g59200
Bradi1g34660	Bradi1g42390
Bradi1g37947	Bradi1g69120
Bradi1g42390	Bradi5g07160
Bradi1g42410	Bradi5g07160

Bradi1g59200	Bradi1g42390
Bradi1g66270	Bradi5g07152
Bradi1g69120	Bradi5g18830
Bradi1g69770	Bradi3g23150
Bradi1g72007	Bradi3g38450
Bradi2g12080	Bradi2g16223
Bradi2g16223	Bradi2g17260
Bradi2g16887	Bradi3g22880
Bradi2g17260	Bradi5g07160
Bradi2g46750	Bradi5g07160
Bradi2g50927	Bradi3g23000
Bradi2g57310	Bradi3g55800
Bradi3g02170	Bradi4g44826
Bradi3g11205	Bradi5g07160
Bradi3g22880	Bradi4g39390
Bradi3g23000	Bradi3g42290
Bradi3g23150	Bradi4g44826
Bradi3g31580	Bradi3g51590
Bradi3g38450	Bradi4g43900
Bradi3g42290	Bradi3g55800
Bradi3g51590	Bradi4g35660
Bradi3g55790	Bradi1g14350
Bradi3g60830	Bradi4g37097
Bradi4g01610	Bradi2g50927
Bradi4g12390	Bradi4g43900
Bradi4g26770	Bradi4g37097
Bradi4g39390	Bradi1g42390
Bradi5g07146	Bradi5g07152

Table S4. List of *BdMATE* genes primers

Primer Name	Sequence (5'–3')	Function
121-BdMATE34_F	TGATTAACAGGGATCCATGTCGTCCCGCTCTGACGCCACG	Clone *BdMATE34*
121-BdMATE34_R	CTGACCCTGAGGATCCGCACTCATCTTTTGGTTGCTCGATCAC	Clone *BdMATE34*
121-BdMATE45_F	TGATTAACAGGGATCCATGGCGGCGACCACGCCGGCTCCGACGAG	Clone *BdMATE45*
121-BdMATE45_R	CTGACCCTGAGGATCCGTTGTCACGTAACTCGGTCTCTGACAAG	Clone *BdMATE45*
BdUBC18_F	GGAGGCACCTCAGGTCATTT	qRT-PCR analysis for reference genes
BdUBC18_R	CGGCAGTTCCTAACATAGCG	qRT-PCR analysis for reference genes
BdMATE02_F	TGGAGGTGGTGAATTTCCC	qRT-PCR analysis for *BdMATE02*
BdMATE02_R	GATCCCCAACCGAGCTTTAAA	qRT-PCR analysis for *BdMATE02*
BdMATE03_F	CATCAACCTCCTCTCCTTCTAC	qRT-PCR analysis for *BdMATE03*
BdMATE03_R	GATCATCATCTCCATGGCACTC	qRT-PCR analysis for *BdMATE03*
BdMATE04_F	GGAAAGGGTTCTCGGTTCTC	qRT-PCR analysis for *BdMATE04*
BdMATE04_R	GATGAGCACTGCAGTGTAGTA	qRT-PCR analysis for *BdMATE04*
BdMATE05_F	ATTTTTCTCAGTTTGGGTCGTG	qRT-PCR analysis for *BdMATE05*
BdMATE05_R	GACGTAGTAACAGCCAATGTTC	qRT-PCR analysis for *BdMATE05*
BdMATE06_F	CAGTGTGAGGGTGAGTAATGAG	qRT-PCR analysis for *BdMATE06*
BdMATE06_R	AGATGTAGCTGATGTAGTCACG	qRT-PCR analysis for *BdMATE06*
BdMATE07_F	GTACATCGTGATGAGCCCC	qRT-PCR analysis for *BdMATE07*
BdMATE07_R	GATGAGCACCTGGAAGTACC	qRT-PCR analysis for *BdMATE07*
BdMATE08_F	GTGCTTCCTGTGTTAGTTCAAG	qRT-PCR analysis for *BdMATE08*
BdMATE08_R	CGATAGTTGTGATGCTCAGTTG	qRT-PCR analysis for *BdMATE08*
BdMATE09_F	GTAAGTTTTGACCGCGTACTTT	qRT-PCR analysis for *BdMATE09*
BdMATE09_R	TCCCCTCCAATTAGCGATTATC	qRT-PCR analysis for *BdMATE09*
BdMATE10_F	GACACATGTTCAAAGGGCTATG	qRT-PCR analysis for *BdMATE10*
BdMATE10_R	CAAGAAGAATTTGGCCAGTGAA	qRT-PCR analysis for *BdMATE10*
BdMATE11_F	CTCTTCACATTCTCCCTCATCC	qRT-PCR analysis for *BdMATE11*
BdMATE11_R	GGAGAGCCATTCTAACTTGGAG	qRT-PCR analysis for *BdMATE11*
BdMATE12_F	CGAAGGTCATGCCATTAATCTG	qRT-PCR analysis for *BdMATE12*
BdMATE12_R	ATGATAAGAAGGCTCCAGTACG	qRT-PCR analysis for *BdMATE12*
BdMATE13_F	CTCTGATGGTCTGCTTAGAGTG	qRT-PCR analysis for *BdMATE13*
BdMATE13_R	AGATGTGTTGAGGGTGATTGAT	qRT-PCR analysis for *BdMATE13*
BdMATE14_F	CGTTTGAGGTTTTGGTGTTACT	qRT-PCR analysis for *BdMATE14*
BdMATE14_R	TTCTTCGCCTTGTCAATGTTAC	qRT-PCR analysis for *BdMATE14*
BdMATE15_F	CAAATCCTAAGCTTGAGGCATC	qRT-PCR analysis for *BdMATE15*
BdMATE15_R	ATACGCCTGACATGATACTGAG	qRT-PCR analysis for *BdMATE15*
BdMATE16_F	CAATGAGCTCACCAAATCGAC	qRT-PCR analysis for *BdMATE16*
BdMATE16_R	AGTAGTAGTGGTCGTAATTGCG	qRT-PCR analysis for *BdMATE16*
BdMATE17_F	CCAATTTTGATGTTCGTTTGCC	qRT-PCR analysis for *BdMATE17*
BdMATE17_R	GGATAACATCAACTTGCTGGAC	qRT-PCR analysis for *BdMATE17*
BdMATE18_F	ATGGTGTTCATACATGGGAGAG	qRT-PCR analysis for *BdMATE18*

BdMATE18_R	GCTCCGATGCAAACAATCAATA	qRT-PCR analysis for *BdMATE18*
BdMATE19_F	CACTTTCGTGGGAAGCTTTTAA	qRT-PCR analysis for *BdMATE19*
BdMATE19_R	TCCAATACCGTATGGGAGATTG	qRT-PCR analysis for *BdMATE19*
BdMATE21_F	GTATCAGTCATGTTCGTTGGC	qRT-PCR analysis for *BdMATE21*
BdMATE21_R	CATGCCAATGAACAAGGTGAAA	qRT-PCR analysis for *BdMATE21*
BdMATE22_F	TGGTGGTCATTTGAGGTCATTA	qRT-PCR analysis for *BdMATE22*
BdMATE22_R	AATTCATTTGCTACTCGAGTGC	qRT-PCR analysis for *BdMATE22*
BdMATE23_F	GAAAATGGTCATGCGGATTGTA	qRT-PCR analysis for *BdMATE23*
BdMATE23_R	GAGCAAAATTGAGCCGAAAATG	qRT-PCR analysis for *BdMATE23*
BdMATE24_F	CAGAGGTCTTCCAGTTCATGAT	qRT-PCR analysis for *BdMATE24*
BdMATE24_R	CAAGACCGTTGCAAGTAGATG	qRT-PCR analysis for *BdMATE24*
BdMATE25_F	TTTCCATGTCGCGTTTAGCTAT	qRT-PCR analysis for *BdMATE25*
BdMATE25_R	CATAGAGAAACATCGTATGCCG	qRT-PCR analysis for *BdMATE25*
BdMATE28_F	GTCATTCTCAGCCTGTATACCA	qRT-PCR analysis for *BdMATE28*
BdMATE28_R	GAATGCAAGCTGGTTGAACTG	qRT-PCR analysis for *BdMATE28*
BdMATE29_F	GAACGTCTCCTACTGGCTTATC	qRT-PCR analysis for *BdMATE29*
BdMATE29_R	ATGCAGCGTAGTACCAAAATTC	qRT-PCR analysis for *BdMATE29*
BdMATE30_F	CCTCCGTTTAGGTTCGAGATTA	qRT-PCR analysis for *BdMATE30*
BdMATE30_R	TAAAGCGTTGATTGGTTGAGTG	qRT-PCR analysis for *BdMATE30*
BdMATE31_F	ATCAACTACCTCTTCGTGACTG	qRT-PCR analysis for *BdMATE31*
BdMATE31_R	GAGGAAGAGAACCAGATTGAGG	qRT-PCR analysis for *BdMATE31*
BdMATE32_F	TTTGCAAGAAGGTTGTAGCATG	qRT-PCR analysis for *BdMATE32*
BdMATE32_R	CCGACAAATATGGGCATTTCAT	qRT-PCR analysis for *BdMATE32*
BdMATE33_F	CCAGGTGGCCTATATTGTGTC	qRT-PCR analysis for *BdMATE33*
BdMATE33_R	TATCAGGAACATGTAGAGCCAG	qRT-PCR analysis for *BdMATE33*
BdMATE34_F	TTCTGGTTCCGCTATGACTTTC	qRT-PCR analysis for *BdMATE34*
BdMATE34_R	CTGCTCTGTTTCGTGTATACCA	qRT-PCR analysis for *BdMATE34*
BdMATE35_F	GATCTGGTACATGATGGTCCTC	qRT-PCR analysis for *BdMATE35*
BdMATE35_R	GTTCAGGTTCATGCAAATGGAG	qRT-PCR analysis for *BdMATE35*
BdMATE36_F	GAACCATTTCGCAGTGATCTTC	qRT-PCR analysis for *BdMATE36*
BdMATE36_R	GAAGAGGTAGCCGAAGATGAAG	qRT-PCR analysis for *BdMATE36*
BdMATE38_F	GCAAGTCATAGTTGCCTATGTC	qRT-PCR analysis for *BdMATE38*
BdMATE38_R	TGTTTTGAACCCTAGGACACAT	qRT-PCR analysis for *BdMATE38*
BdMATE39_F	CTCTTGGAGCAATTCTAGGCTA	qRT-PCR analysis for *BdMATE39*
BdMATE39_R	AACCGCTCCTGAGAAATCTTTA	qRT-PCR analysis for *BdMATE39*
BdMATE40_F	GATGGACATCGTTGGTATACCT	qRT-PCR analysis for *BdMATE40*
BdMATE40_R	TGCTGCAAGTGATACTATGACA	qRT-PCR analysis for *BdMATE40*
BdMATE41_F	CTACATCCTCTGCTCGCTC	qRT-PCR analysis for *BdMATE41*
BdMATE41_R	GACGAGGACGAAGTTGATGG	qRT-PCR analysis for *BdMATE41*
BdMATE42_F	GTCTCAGACTTCTCATACTCGG	qRT-PCR analysis for *BdMATE42*
BdMATE42_R	AGATCTTTCTGGTGCAAAAACC	qRT-PCR analysis for *BdMATE42*
BdMATE43_F	CTTCTCCATGGAGGGGTTTAAG	qRT-PCR analysis for *BdMATE43*

BdMATE43_R	TTCAAAAGCCCAAAACTCTACG	qRT-PCR analysis for *BdMATE43*
BdMATE44_F	CTGTGGTACAACAGCATATTGG	qRT-PCR analysis for *BdMATE44*
BdMATE44_R	GCATATTGAAAGAGCGTCAAGT	qRT-PCR analysis for *BdMATE44*
BdMATE45_F	ATCTTGTCAGAGACCGAGTTAC	qRT-PCR analysis for *BdMATE45*
BdMATE45_R	CTATTTTTGACAGGGTTCACCG	qRT-PCR analysis for *BdMATE45*
BdMATE48_F	GTGGTTGCAATAATCTGGGTTT	qRT-PCR analysis for *BdMATE48*
BdMATE48_R	GATCATCCACCGGATATAGCTC	qRT-PCR analysis for *BdMATE48*
BdMATE49_F	CTTCTGGTTCCACTTCGACTTC	qRT-PCR analysis for *BdMATE49*
BdMATE49_R	GATGTCTGCGTCGATAAGTAAG	qRT-PCR analysis for *BdMATE49*
